# Clinical and Pathological Findings Associated with Aerosol Exposure of Macaques to Ricin Toxin

**DOI:** 10.3390/toxins7062121

**Published:** 2015-06-09

**Authors:** Seth H. Pincus, Manoj Bhaskaran, Robert N. Brey, Peter J. Didier, Lara A. Doyle-Meyers, Chad J. Roy

**Affiliations:** 1 Departments of Pediatrics and Microbiology, Louisiana State University School of Medicine, Children’s Hospital, 200 Henry Clay Ave, New Orleans, LA 70118, USA; 2 Lilly Research Laboratories, 355 East Merrill Street, Indianapolis, IN 46285, USA; E-Mail: bhaskaran_manoj@lilly.com; 3 Kinesis Vaccines, Chicago, IL 60030, USA; E-Mail: rnbrey@kvax.org; 4 Divisions of Pathology, Veterinary Medicine, and Microbiology, Tulane National Primate Research Center, 18703 Three Rivers Road, Covington, LA 70433, USA; E-Mails: pjdidier@tulane.edu (P.J.D.); ldoyle@tulane.edu (L.A.D.-M.); croy@tulane.edu (C.J.R.); 5 Department of Microbiology and Immunology, Tulane School of Medicine, 1430 Tulane Ave., New Orleans, LA 70112, USA

**Keywords:** ricin, toxin, aerosol exposure, syndromic diagnosis, biodefense, telemetric monitoring

## Abstract

Ricin is a potential bioweapon that could be used against civilian and military personnel. Aerosol exposure is the most likely route of contact to ricin toxin that will result in the most severe toxicity. Early recognition of ricin exposure is essential if specific antidotes are to be applied. Initial diagnosis will most likely be syndromic, *i.e.*, fitting clinical and laboratory signs into a pattern which then will guide the choice of more specific diagnostic assays and therapeutic interventions. We have studied the pathology of ricin toxin in rhesus macaques exposed to lethal and sublethal ricin aerosols. Animals exposed to lethal ricin aerosols were followed clinically using telemetry, by clinical laboratory analyses and by post-mortem examination. Animals exposed to lethal aerosolized ricin developed fever associated with thermal instability, tachycardia, and dyspnea. In the peripheral blood a marked neutrophilia (without immature bands) developed at 24 h. This was accompanied by an increase in monocytes, but depletion of lymphocytes. Red cell indices indicated hemoconcentration, as did serum chemistries, with modest increases in sodium and blood urea nitrogen (BUN). Serum albumin was strikingly decreased. These observations are consistent with the pathological observations of fluid shifts to the lungs, in the form of hemorrhages, inflammatory exudates, and tissue edema. In macaques exposed to sublethal aerosols of ricin, late pathologic consequences included chronic pulmonary fibrosis, likely mediated by M2 macrophages. Early administration of supportive therapy, specific antidotes after exposure or vaccines prior to exposure have the potential to favorably alter this outcome.

## 1. Introduction

Ricin is a plant cytotoxin that poses a significant risk as a biothreat, primarily as a toxin that could be delivered by aerosolization in acts of bioterrorism and biological warfare [1,2,3,4,5,6,7]. Ricin is an A/B toxin derived from the castor bean of *Ricinus communis*, which is cultivated for the oil extracted from the beans, but also grows as a weed throughout the temperate and tropical zones. Ricin toxin can constitute up to 5% of the total protein of the castor bean and can be extracted from the mash produced as a by-product of castor oil production by several simple enrichment steps. Because of the copious amounts of processed castor beans and the high content of ricin, it may be possible to purify over 50,000 tons of pure ricin per year, and hence the overall concern of its potential as a bioweapon in a pure or impure form [8]. The toxin is also quite stable. There have been documented individual murders using ricin. In the United States alone, ricin events of differing seriousness have occurred, ranging from attempts by disgruntled individuals to organized plots meeting the definitions of terrorism. Ricin has become the “poor man’s toxin” of choice, popularized by the television series Breaking Bad. Effectively delivering toxin is the major limitation to its use as a weapon of mass terror. Accumulating evidence that oral toxicity of ricin is limited (at least in mice) eases concerns of food or water contaminations as a route of attack [9,10]. Effective aerosol delivery of ricin requires the generation of micron-sized particles to reach the distal lung, as well as to remain suspended in the air for sufficient time to produce a mass exposure [9,11,12,13]. This requires a degree of technical proficiency on the part of a terrorist, but certainly could be done without arousing suspicion.

Ricin is a promiscuous toxin, binding to multiple glycans on the surfaces of all cell types, through the galactose-binding of the lectin B chain [14,15]. The clinical syndrome is thus dependent on the route of exposure. Because the gravest bioterrorist threat of attack with ricin lies in mass respiratory exposure, we believe it particularly important to understand the clinical syndrome associated with exposure via this route, so that we can diagnose it early and treat it better. Murine studies have clearly demonstrated the importance of both systemic and pulmonary inflammation in mediating the toxic effects following aerosol exposure [16,17,18,19]. In macaques, there is clear histological evidence of pulmonary inflammation at necropsy following both acute lethal toxicity and sub-acute chronic lung disease [9,11,20]. However disease manifestations observed in macaques, including clinical observations, physical signs and laboratory abnormalities, are not fully described. We do so in this manuscript, and also describe the underlying pathogenesis by linking disease manifestations to post-mortem histological findings.

## 2. Results and Discussion

### 2.1. Lethal Aerosolized Ricin Exposure

To understand the clinical, laboratory, and pathologic outcome of exposure to ricin toxin via aerosol inhalation, we have studied the effects of ricin in six rhesus macaques, four of which had implanted telemetry devices. These macaques were used as unvaccinated control individuals in studies to examine the efficacy of a ricin A chain vaccine [21]. Male macaques were exposed to ricin toxin in micron-sized (1.2 µm MMAD, 1.4 σ_g_) aerosols for 10 min, and dosage calculated by predictive plethsymography, as described elsewhere [9,11,12,21]. The characteristics of each animal are shown in Table 1. Doses varied (ranging from 1.6 to 6.6 LD_50_, LD_50_ = 5.8 µg/kg) due to differences in respiratory rates and depth of respiration among macaques. Animals were euthanized based upon pain assessment guidelines and necropsies were performed on these animals 25–52 h following lethal challenge.

**Table 1:** Characteristics of macaques used in these studies.

ID	Expt	Age (year)	Telemetry	Weight (kg)	Dose (µg/kg)	Survival (h)	% weight change	Lung % body weight
JE27	1	4.16	N	7.99	38.5	25	−2.63	1.47
JL68	1	3.71	Y	4.98	26.8	27	−1.81	1.94
KA65	1	2.16	Y	3.49	9.4	28	1.43	2.06
JD11	2	4.34	Y	5.81	13.9	52	−8.78	2.34
JR16	2	3.24	Y	4.56	29.6	46	−10.09	2.89
KI03	2	2.1	N	3.81	22.6	48	−4.46	2.77

There was a clear difference in survival time between Experiment 1 and Experiment 2. Time of euthanasia was determined by the veterinarian, in consultation with animal care staff. The same individuals carried out Experiments 1 and 2. There was little difference in the initial characteristics of the animals, nor the doses they received. The animals in Experiment 2 lost more weight (−7.78 *vs.* −1.0 kg, *p* = 0.032) and had larger lungs (2.67 *vs.* 1.82% of total body weight, *p* = 0.026, normal is <0.8% of body weight), suggesting that the Experiment 2 animals were sicker at the time of euthanasia, perhaps a function of the greater time post exposure allowing for greater changes.

### 2.2. Clinical Signs Following Lethal Ricin Exposure

**Figure 1:**
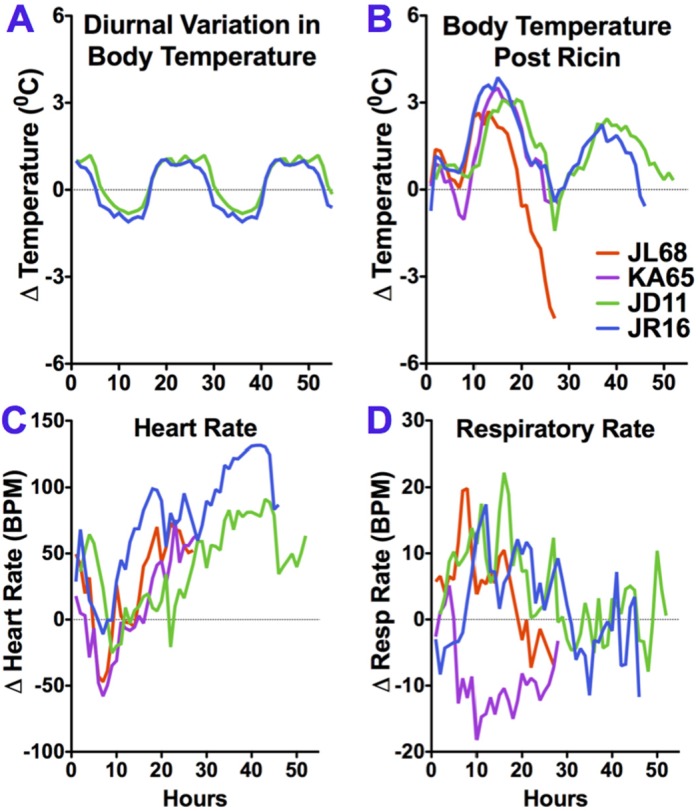
Telemetric monitoring of vital signs following aerosol exposure to ricin toxin. Changes in the mean hourly values for the parameters are plotted against time for each animal. Alterations in body temperature in non-exposed macaques, showing normal diurnal variation (**A**), and following ricin exposure (**B**); heart rate (**C**) and respiratory rate (**D**) are shown. The fever (**B**) and heart rate (**C**) curves, shown here as individual animals, were averaged and shown as the controls in Figure 2 of reference [21].

Four of the animals were followed by telemetry to measure body temperature, respiratory rate, and cardiac rate. Changes in these parameters were plotted *versus* time (Figure 1). Temperature curves are shown at the top. On the left is the normal diurnal variation observed in healthy animals before exposure, on the right the effects of ricin exposure are shown. Ricin elicited both hyperthermia and temperature instability. A small spike in temperature 2–3 h post exposure was followed by a longer and higher fever, beginning 9–12 h after exposure. The abrupt fall in body temperature starting at 20 h was associated with anesthesia and bleeding. This is evidence of a failure of temperature regulation, since there is no evidence of a similar fall in temperature in the pre-exposure animals who underwent the same procedure. For the animals in Experiment 1, this instability resulted in a terminal fall in body temperature. Those in Experiment 2 had a rebound in fever, before the premorbid temperature loss. Heart rate dipped 6–10 h post exposure, then steadily climbed before dropping in the hours before death. Surprisingly, respiratory rate seemed least effected. Three animals had a somewhat increased respiratory rate for the first day post exposure, while one had an almost mirror dip. Interestingly respiratory rates in all animals returned to normal thereafter.

All animals were monitored frequently by veterinarians and animal technicians, and received a full physical examination while under anesthesia at 22–24 h. At this time, animals displayed pallor of the mucous membranes, wheezing and dyspnea. In one animal, muffled heart sounds suggested the possibility of pericardial effusion.

### 2.3. Laboratory Findings

**Figure 2:**
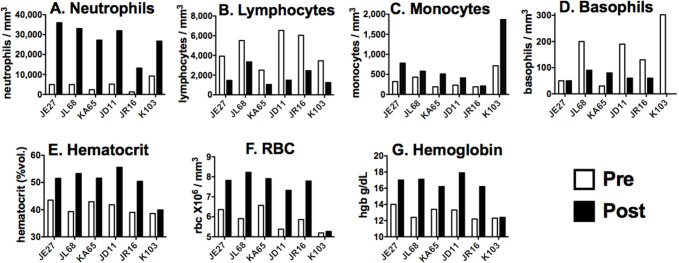
Hematologic changes in individual macaques. White cell counts (**A**–**D**) and red cell indices (**E**–**G**) are compared prior to and 20–22 h following ricin exposure.

**Figure 3:**
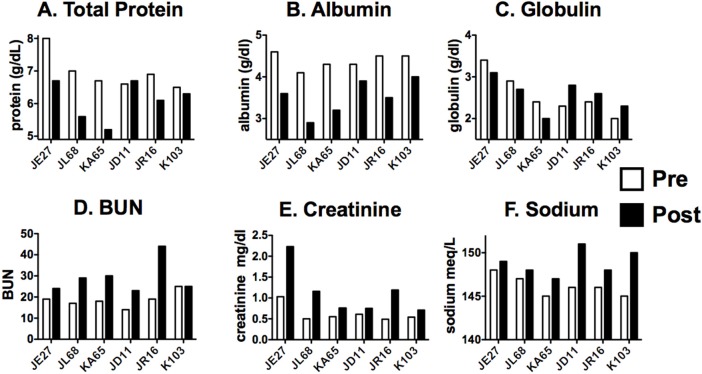
Changes in selected clinical chemistry values in individual macaques. Serum protein concentrations are shown at (**A**–**C**), parameters demonstrating possible hemoconcentration on the (**D**–**F**).

Complete blood counts with differential (CBC) and serum chemistry values were measured in the clinical pathology laboratory prior to and 20–22 h following exposure to ricin toxin. Table 2 and Figure 2 show changes observed in CBC; Table 3 and Figure 3 show clinical chemistries. The tables show the mean changes and statistical significance of all values. The figures show pre and post values of individual monkeys for selected laboratory parameters.

**Table 2:** Changes in complete blood count following exposure to ricin.

Parameter	Units ^1^	Δ Post Ricin		
		Mean	SEM	P ^2^
Neutrophil	cell/mm^3^	24192.8	3301.4	0.0005
Lymphocyte	cell/mm^3^	−1904.2	1166.9	0.0031
Monocyte	cell/mm^3^	452.2	161.9	0.0709
Eosinophil	cell/mm^3^	106.3	139.0	0.6195
Basophil	cell/mm^3^	37.1	15.5	0.0573
Platelets	platelets/mm^3^	−333.3	17742.0	0.9857
RBC	cell × 10^6^/mm^3^	1.51	0.32	0.0053
Hgb	g/dL	3.20	0.70	0.0060
Hct	% by volume	9.53	1.94	0.0044

^1^ Units are traditional units as used in USA; ^2^ P by two-tailed, paired (pre and post-exposure) student’s *t* test.

Abnormalities were observed in both the WBC and RBC compartments (Table 2, Figure 2). There is a marked increase in the number of neutrophils, with mean values increasing >6×. Interestingly, there were bands in only one animal, and no evidence of other immature WBC. This suggests that the increase in circulating neutrophils is not due increased production, but to desequestration, probably by demarginating from the walls of blood vessels. Dohle bodies were observed in the neutrophils of some animals. These tangles of rough endoplasmic reticulum are found in inflammatory states. There were more modest increases in the number of monocytes (a mean of 2× in 6 of 6 animals) and basophils (3× in 5 of 6 animals), neither of which was statistically significant. Interestingly, there was a significant decrease in the number of lymphocytes, observed in all animals, consistent with the lymphoid depletion observed in peribronchial lymph nodes at necropsy in macaques exposed by aerosol [11], and in mice receiving systemic doses of ricin [9]. Platelet counts were unaffected, surprisingly because platelet counts are considered an acute phase reactant, a marker of acute inflammation. All of the red cell indices were increased, although there was no change in the mean corpuscular volume (not shown), consistent with hemoconcentration.

Serum chemistry values were remarkably unaltered during this acute inflammatory crisis (Table 3, Figure 3). Sodium, BUN, and creatinine were moderately increased. Again hemoconcentration is one explanation. Mildly decreased renal function may result from fluid shifting from the circulatory compartment. There was a marked decrease in circulating albumin, observed in all animals. Globulin levels were unchanged overall, but there was a statistically significant difference between the animals in Experiment 1 and 2 (*p* = 0.004, two tailed, unpaired Student’s t test), with animals in 1 having decreased globulin, but increased in Experiment 2. It seems unlikely that this difference would account for the increased time of survival in these animals. There was no evidence of hypoglycemia, although this has been reported as the primary cause of death in mice systemically injected with ricin [9,22]. We do not know if this difference is due to the species or route of administration. There were mild elevations in the hepatic transaminases. Other than glucose, all blood chemistry values were consistent with those previously reported in mice [22].

Taken together, these results are consistent with an acute inflammatory response, but other than neutrophilia, there were surprisingly few systemic signs. There was evidence of hemoconcentration from changes in red cell indices as well as of BUN and sodium, although it should be noted that there was no correlation among macaques in the magnitude of change in RBC values and serum chemistries. As will become clear in the next section, hemoconcentration resulted from large amounts of fluid being sequestered in the lung cavities and pleural cavity. For all of this, one day following ricin exposure there was little evidence in the serum chemistries of renal or hepatic failure.

**Table 3:** Changes in serum chemistry values 20–22 h following exposure to ricin.

Parameter	Units	Δ Post Ricin		
		Mean	SEM	P
Na	mEq/L	2.67	0.76	0.0171
K	mEq/L	0.55	0.32	0.1475
Cl	mEq/L	0.67	1.43	0.6606
Glucose	mg/dL	14.33	7.31	0.1070
BUN	mg/dL	10.50	3.45	0.0287
Creatinine	mg/dL	0.51	0.17	0.0300
Total protein	g/dL	−0.85	0.27	0.0268
Albumin	g/dL	−0.87	0.14	0.0014
Globulin	g/dL	0.02	0.15	0.9154
AST	U/L	25.33	12.07	0.0898
ALT	U/L	4.00	2.77	0.2082

### 2.4. Results of Post-Mortem Examinations

**Figure 4:**
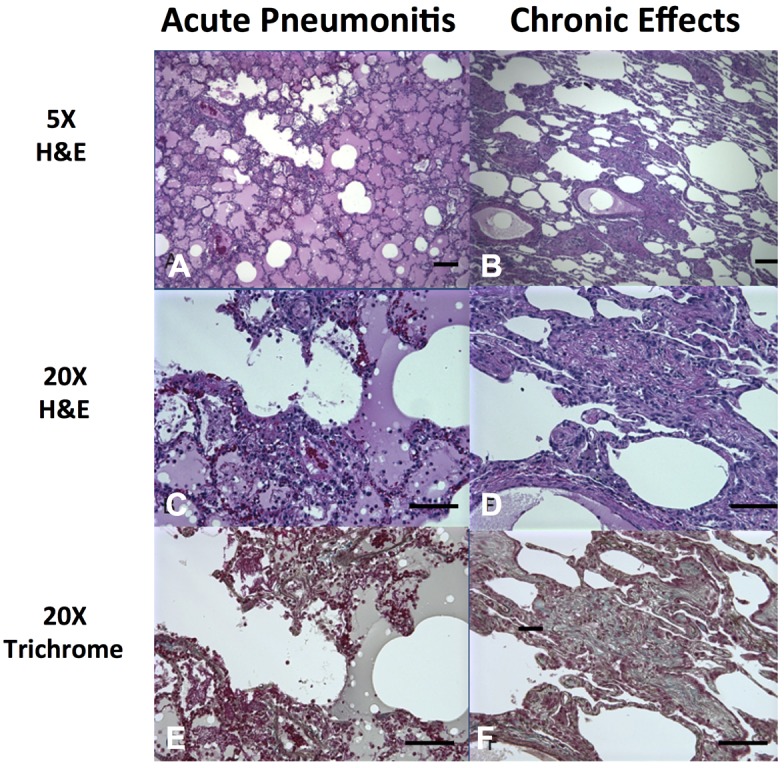
Histological examination of lung tissue one day following exposure to lethal (acute) and 20 days following sublethal (chronic) concentrations of ricin toxin. Acute changes were characterized by massive alveolar edema, infiltrations of mixed inflammatory cells and marked alveolar necrosis (**A**,**C**,**E**). Chronic changes were characterized by areas of interstitial fibrosis which frequently obliterate functional alveolar spaces (**B**,**D**,**F**). Scale bars in the lower right corner of each figure represent 300 µm (**A**,**B**) or 50 µm (**C**–**F**).

All animals underwent pathological examination following euthanasia. Abnormal pathology was confined to the chest. Lungs and lymph nodes were grossly enlarged, with obvious areas of hemorrhages, overall edema and pleural effusions. The lung weights were increased 2–3× over that of healthy lungs (Table 1). There was a large fluid shift to the lungs in the form of hemorrhages, inflammatory exudates, and tissue edema. This represents a shift of 18.5% ± 2.8% the total blood volume into the lungs of animals necropsied one day following exposure (Experiment 1), and 31.6% ± 2.6% at two days (Experiment 2). Microscopic examination (Figure 4, left panel) reveals eosinophilic effusions and fibrin deposition in many alveolar, bronchiolar, and bronchial air spaces with scattered necrosis of bronchiolar epithelium mixed with inflammatory cellular infiltrates that include many neutrophils, as has been described in detail elsewhere [11]. Respiratory failure induced by this intense inflammatory pneumonitis would almost certainly be the ultimate cause of death. Monitoring blood pressure and oxygenation in future studies should clarify the terminal events.

### 2.5. Sublethal Aerosolized Ricin Exposure

**Figure 5:**
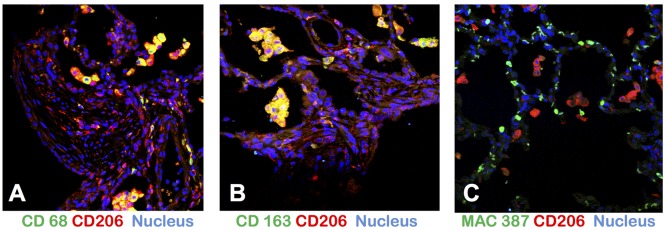
Confocal microscopy to identify subset of macrophages found within the intra-alveolar space 20 days following sublethal exposure to aerosolized ricin. Co-expression of CD206, the mannose receptor (red with CD68, CD163, or MAC387 were visualized to see the changes in the distribution of specific subsets of macrophages in relation to their location in lung. Almost all intra-alveolar macrophages co-expressed CD206 with CD68 and CD163 (orange signal in **A** and **B**). MAC387 (green signal panel **C**) was present exclusively in the interstitial/intravascular macrophages and was absent in which CD 206+ intra-alveolar macrophages. Cell nuclei were stained blue with DAPI.

Animals that were exposed to sublethal concentrations of ricin (~3 µg/kg) in preliminary dosing studies were sacrificed 11–20 days post exposure. This part of the evaluation of ricin toxicity was done to examine the effects of ricin in the lungs of animals that survived ricin exposure, as a potential model for the long term effects of ricin in humans that survive aerosol ricin exposure. The results (Figure 4B,D,F) were similar to those described in greater detail elsewhere [11], including extensive fibrosis, infiltrates containing PMNs and their debris, and many macrophages, including foamy macrophages within the alveolar spaces. Confocal microscopy was used to further characterize the macrophages infiltrating the persistent lesions (day 20). The results are shown in Figure 5. CD206 expression was used to identify the broader class of macrophages, while CD68, CD163, and MAC 387 expression was used to identify subsets of macrophages [23,24,25]. The majority of the intra-alveolar macrophages, described as large and foamy, appear to be of the anti-inflammatory M2 subtype [25]. CD206 was coexpressed with both CD68 and CD163 on the large majority of these cells. However, MAC387 and CD 206 expression was mutually exclusive and MAC387 expression was present exclusively in the interstitial macrophages and was completely absent in the intra-alveolar macrophages. MAC387 is believed to be a marker for early stages of tissue infiltration in certain inflammatory or immune states [24]. The role of these cells in the long-term evolution of chronic disease, as might be observed in humans who almost certainly will survive a ricin attack, should be a matter of study, and may point to pharmacologic targets to modify long-term pathogenesis [25].

## 3. Experimental Details

Animal husbandry was exactly as described elsewhere [11,21]. Rhesus macaques (*Macaca mulatta*) of Indian origin were born and bred at the Tulane National Primate Research Center (TNPRC; Covington, LA, USA), which is a U.S. Department of Agriculture (USDA) licensed and fully accredited by the Association for Assessment and Accreditation of Laboratory Animal Care (AAALAC). Animals were singly housed and fed primate chow (Harlan Teklad, Madison, WI, USA) and water ad libitum. Animals were directly observed a minimum of twice daily for clinical changes. Animals determined to be in sufficient respiratory distress to cause pain or suffering (a decision made by the animal support staff in consultation with the attending veterinarian) and those that survived until the end of each trial were euthanized by an overdose of sodium pentobarbital, consistent with the recommendation of the American Veterinary Medical Association’s (AVMA) Panel on Euthanasia, and submitted for necropsy. All methods were approved by the Tulane Institutional Animal Care and Use Committee (IACUC). This study was performed as a part of several independent studies to evaluate a ricin toxin post-exposure therapeutic compound and the effects of vaccination [21]. Animals used in the current evaluation represented the untreated controls for lethal and sublethal ricin exposure. Prior to the experiment, subcutaneous radiotelemetry transmitters were implanted in four anesthetized animals using aseptic surgical technique, as described [21]. Post mortem studies were performed on animals euthanized 28–48 h following exposure to lethal doses of ricin, and 20 days after a sublethal dose [11,21].

Ricin aerosol exposures were performed within the infectious disease aerobiology laboratories at the TNPRC. The procedures have been described elsewhere [9,11,12,21]. Ricin was produced at the University of Texas Southwestern [26] and was obtained from BEI resources. Inductive plethysmography was twice performed on each animal: To measure respiratory rate and volume, two days prior to exposure to for dosage preparation, and immediately prior to exposure for calculation of administered dose. Aerosol exposure was performed in a head-only chamber. The target dose was 3.3 LD_50_ (LD_50_ = 5.8 mg/kg). Ricin was dissolved in 10 mL PBS and nebulized over 10 min at 7.5 L/min producing 3 × 10^4^ particles/cc with a mass median aerodynamic diameter of ~1.4 µm. Aerosols were sampled continuously to measure ricin concentrations. The inhaled dose was calculated multiplying the experimentally determined ricin aerosol concentrations by the volume of air inhaled (determined by plethysmography). Animals were examined twice daily. Blood was sampled prior to and 20–22 h post-exposure. Animals were anesthetized for this procedure. Automated complete blood counts and chemistry analyses were performed using best clinical practices and standard laboratory protocols. All animals underwent full necropsy and pathological examination. Methods for tissue processing and staining have been described in detail elsewhere [11]. Zinc/formalin-fixed, paraffin embedded tissues were stained with either hematoxalin and eosin, or Gamori’s one-step trichrome stain.

Tissue samples for confocal microscopy were obtained from paraffin blocks. 5 µm tissue sections were mounted on slides, baked overnight at 56 °C and passed through xylene, graded ethanol, and double distilled water to remove paraffin and rehydrate tissue sections. Heat-induced epitope retrieval was performed by microwaving, followed by boiling for 20 min in citrate solution pH 6.0. Slides were stained after blocking with 10% normal goat serum and 0.02% fish skin gelatin (Sigma Chemical, St. Louis, MO, USA, #G7765). Individual slides were incubated with murine IgG1 mAbs to CD68 (Dako, Carpintaria, CA, USA, catalogue #M0814), CD163 (ABD Serotec, Raleigh, NC, USA, #MCA1853), and MAC387 (Dako #M0747), washed, and incubated with secondary goat anti-mouse IgG1 conjugated to Alexa Fluor 488. Following incubation and washes, the staining process was then repeated with rabbit anti-CD206 (Sigma #HPA004114) and goat anti-rabbit IgG conjugated to Alexa Fluor 568. Slides were mounted and allowed to dry overnight before imaging with a Leica TCS SP2 confocal microscope (Wetzler, Germany) equipped with three lasers: An argon-krypton 488 nm laser, a DPSS 561 nm laser and a helium-neon 633 nm laser.

## 4. Conclusions

Exposure of rhesus macaques to lethal doses of aerosolized ricin resulted in an acute syndrome of fever and temperature instability, increasing respiratory distress associated with an inflammatory pneumonia, accompanied by laboratory and pathological evidence of profound fluid shifts. Respiratory failure was the most likely cause of death, which ensued within 28–48 h after aerosol exposure. On the other hand, exposure to sublethal doses of aerosolized ricin resulted in recovery from acute lung disease, but surviving animals developed signs of long-term injury [11]. Most humans are likely to survive a ricin attack, due either to low dose exposure resulting from ineffective aerosolization, or because of effective treatment, primarily supportive [27,28]. Vaccination with ricin A chain subunit immunogen may allow prevention of the major toxicity of aerosolized ricin. In studies associated with the current evaluation, macaques vaccinated three times with ricin A chain vaccine adsorbed to aluminum adjuvant were protected against aerosolized ricin [21]. In the protected macaques lungs were grossly normal after ricin exposure, except for occasional mild multifocal proliferation of fibroblasts with collagen deposition around terminal respiratory bronchioles, suggesting resolving epithelial damage and an ongoing immune response. Therefore, study of the chronic effects of ricin exposure and its mitigation will be important in the development of vaccines and therapeutics for ricin exposure. In this regard, our observation that M2 macrophages appear to be the predominant cell type found within the alveolar space may be crucial. These cells secrete anti-inflammatory cytokines (e.g., IL-10 and IL-1RA), promote wound healing, and tissue remodeling, but may also be associated with fibrosis and promote tumor progression [25,29,30]. M2 macrophages arise in response to M-CSF, the T_H_2 cytokine IL-4, and chemokines CCL2 and CXCL10. The role of these cells in the long-term evolution of chronic pathology may point to pharmacologic targets for limiting post-inflammatory lung damage.

The predilection of ricin to cause lymphocyte depletion is not fully explained and the immunologic consequences unknown. Here we observed a decrease in the numbers of circulating lymphocytes (Table 2). Previous studies of macaques exposed to aerosolized ricin not only showed a marked cellular depletion in the bronchial lymph nodes, with destruction of the architecture, but also showed evidence of a more systemic depletion [11]. Similar patterns of lymphocyte depletion were observed following systemic administration of ricin to mice [9]. A potential anatomic explanation is that lymphocytes circulate and are more likely to encounter ricin. For this to fully explain the evidence of systemic effects, one would need to postulate that the lymphocytes themselves are involved in the trafficking of ricin to distant lymph nodes and subsequent depletion of lymphocytes. An alternative explanation is that lymphocytes are more sensitive to ricin than other cell types. Such sensitivity could be explained by higher levels of cell-surface glycans, or higher rates of internalization of the glycans, similar to the causes of differential susceptibility in ricin-resistant variant cells [31].

The information presented here may be of use to physicians seeking to provide supportive therapy to victims of aerosolized ricin. The animals reported here served as controls in a larger study to determine the protective efficacy of a ricin vaccine [21]. Although that vaccine provided complete protection from the lethal effects of exposure, pathological examination revealed evidence of chronic inflammation 14 days post exposure. But active immunization will only be used with foreknowledge of weaponized ricin. Administration of specific post-exposure antidotes, such as passive immunization with mAbs [10,28,32,33], is likely to be delayed until well after the onset of symptoms. Here we have provided important information that may be used by clinicians for early recognition of aerosol toxin exposure, and the application of supportive therapies to manage the hemodynamics of the fluid shifts that accompany such exposure.
